# Metastatic colorectal cancer to a primary thyroid cancer

**DOI:** 10.1186/1477-7819-6-122

**Published:** 2008-11-11

**Authors:** Martin H Cherk, Maggie Moore, Jonathan Serpell, Sarah Swain, Duncan J Topliss

**Affiliations:** 1 Department of Nuclear Medicine, the Alfred Hospital, Commercial Road, Melbourne Victoria 3004, Australia; 2 Department of Medical Oncology, the Alfred Hospital, Commercial Road, Melbourne Victoria 3004, Australia; 3 Department of Surgery, the Alfred Hospital, Commercial Road, Melbourne Victoria 3004, Australia; 4 Department of Anatomical Pathology, the Alfred Hospital, Commercial Road, Melbourne Victoria 3004, Australia; 5 Department of Endocrinology and Diabetes, the Alfred Hospital, Commercial Road, Melbourne Victoria 3004, Australia

## Abstract

**Background:**

Metastatic malignancy to the thyroid gland is generally uncommon due to an unfavourable local thyroid micro-environment which impairs the ability of metastatic cells to settle and thrive. Metastases to the thyroid gland have however been reported to occur occasionally particularly if there has been disruption to normal thyroid tissue architecture.

**Case presentation:**

We report a patient with a history of surgically resected rectal adenocarcinoma who presents with a rising serum CEA level and an ^18^F-FDG PET scan positive thyroid nodule which was subsequently confirmed at surgery to be a focus of metastatic rectal adenocarcinoma within a primary poorly differentiated papillary thyroid carcinoma.

Subsequent treatment involved right hemi-thyroidectomy, pulmonary wedge resection of oligometastatic metastatic colorectal cancer and chemotherapy.

**Conclusion:**

Metastatic rectal carcinoma to the thyroid gland and in particular to a primary thyroid malignancy is rare and unusual. Prognosis is likely to be more dependent on underlying metastatic disease rather than the primary thyroid malignancy hence primary treatments should be tailored towards treating and controlling metastatic disease and less emphasis placed on the primary thyroid malignancy.

## Background

Metastatic malignancy to the thyroid gland, although considered rare, occurs more frequently than expected. Microscopic metastases to the thyroid gland have been reported to occur in 4%–9% of autopsy studies[1,2], with breast, lung, melanoma and kidney the most common primary malignancies. Metastatic colorectal cancer to the thyroid gland is considered unusual, with 33 previous cases reported in the literature up till 2008 [3-6]. There are no previously published reports of metastatic malignancy to a primary thyroid malignancy.

## Case presentation

**Figure 1:**
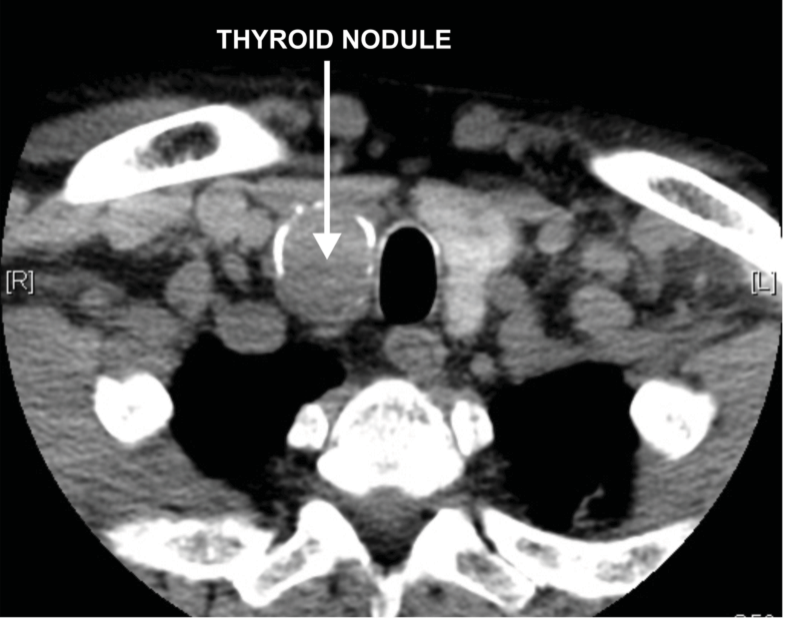
CT scan demonstrating 4.3 × 2.5 cm partially calcified complex dominant nodule in the inferior pole of the right lobe of thyroid.

A 52 year old man with a history of T3N1M0 (Dukes C) rectal adenocarcinoma treated with neo-adjuvant chemo-radiotherapy (5FU + radiotherapy) followed by anterior resection and adjuvant chemotherapy (5FU) presented 18 months post completion of therapy with rising serum CEA level (2.3 μg/l post resection of primary rectal malignancy to 9.7 μg/l) and a 15 mm left lower lobe pulmonary nodule on computerized tomography (CT), suggestive of a metastatic deposit (figure 1). He had no other significant past medical history and no family history of malignancy. Physical examination was unremarkable, with no obvious mass lesions in the abdomen or palpable local recurrence in the rectal stump.

**Figure 2:**
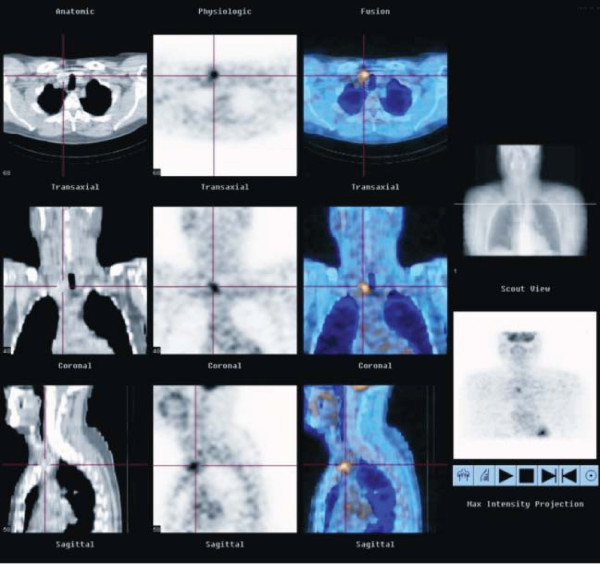
^18^**F-FDG PET/CT scan demonstrating focal intense **^18^**F-FDG uptake in the nodule in the inferior pole of the right lobe of thyroid.**

An ^18^F-FDG whole body Positron Emission Tomography (PET) scan was performed to further evaluate the nature of the left lower lobe pulmonary nodule and to evaluate any other possible sites of metastatic disease. The whole body PET scan demonstrated significant ^18^F-FDG uptake in the left lower lobe pulmonary nodule compatible with a metastatic deposit (figure 2). An intensely FDG-avid right lower lobe thyroid nodule was also noted, which corresponded with a large partially calcified well circumscribed nodule on CT.

**Figure 3:**
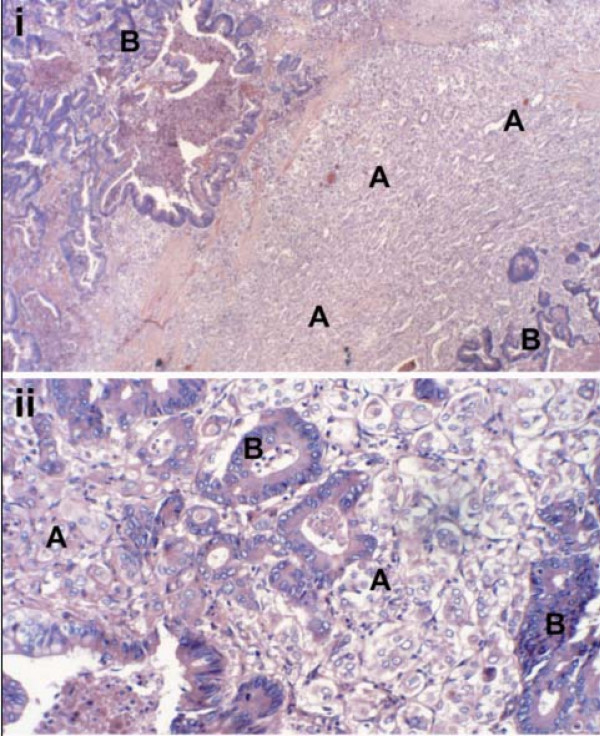
Histological examination of the thyroid nodule revealed (A) poorly differentiated primary papillary thyroid carcinoma intermixed with (B) foci of metastatic rectal adenocarcinoma i H&E Low power ×10 ii H&E High Power ×20.

An ultrasound guided fine needle aspirate of the right lower pole thyroid nodule was performed which revealed malignant cells with features suggestive of a primary papillary thyroid cancer. A right hemi-thyroidectomy was subsequently performed. Macroscopically, the resected thyroid specimen demonstrated a well-circumscribed dominant thyroid nodule measuring 32 mm in diameter with a pale tan capsule less than 1 mm in thickness. The cut surface had a variegated appearance with pale tan friable tissue intermixed with foci of yellow tissue and dark brown foci. Histological examination revealed cells typical of metastatic adenocarcinoma of the colon intermixed in a background of a poorly differentiated papillary thyroid carcinoma (figure 3).

**Figure 4:**
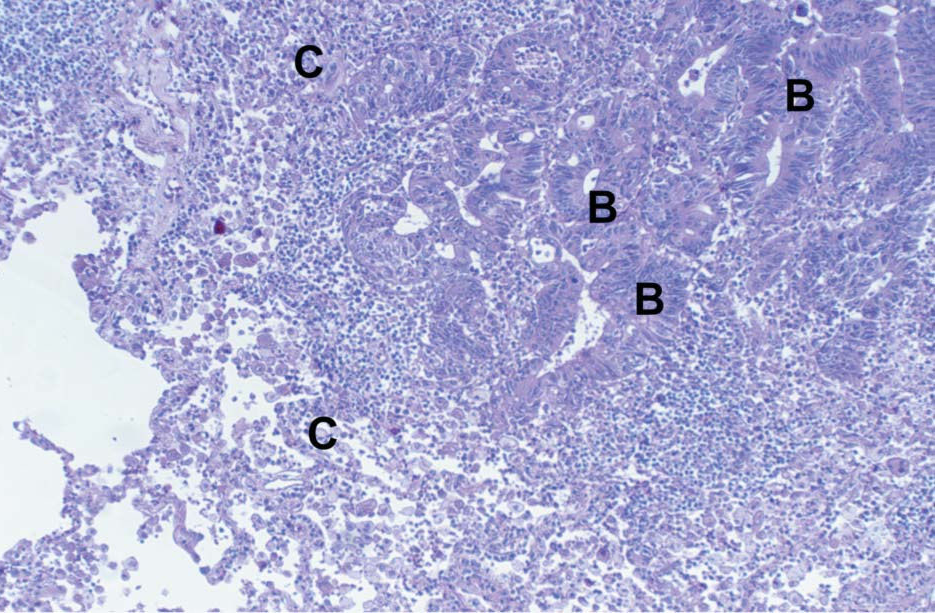
Histological examination of the resected pulmonary nodule revealed (B) metastatic rectal adenocarcinoma in a background of (C) normal lung parenchyma. H&E High Power ×20.

A wedge resection of the left lower lobe pulmonary nodule was subsequently performed which confirmed metastatic colorectal adenocarcinoma (figure 4). Serum CEA level normalized post operatively (1.9 μg/l) and no further surgery was contemplated. Despite no further overt metastatic disease on CT, the patient was commenced on a course of chemotherapy (5 FU/Oxaliplatin) to treat presumed low volume metastatic colorectal disease and thus decrease the risk of developing overt recurrence. No further thyroid cancer specific treatment has been initiated.

## Discussion

Metastatic lesions to the thyroid gland are generally considered rare, possibly due to a high oxygen and iodine environment which may impair the ability of metastatic cells to settle and develop. Abundant high velocity blood flow through the thyroid gland also possibly plays a role in impeding the ability for metastatic cells to gain a foothold[3]. Perhaps unsurprisingly, when primary thyroid pathology occurs which results in structural change, this has been associated with an increased incidence of metastases to the thyroid gland. Multinodular goiters and adenomatous change have both been associated with an increased incidence of metastases to the thyroid gland[7]. It is possible our patient had a pre-existing primary thyroid carcinoma at the time of initial surgery for the primary rectal malignancy which altered the local thyroid environment rendering conditions more favorable for metastatic rectal adenocarcinoma cells to settle.

With the advent of improved diagnostic imaging technology such as ^18^F-FDG PET, an increasing number of incidental cases of metastatic disease to the thyroid gland are likely to be detected. Well differentiated primary thyroid carcinomas such as papillary and follicular carcinomas are generally not ^18^F-FDG avid on PET scanning and usually only become ^18^F-FDG avid if they de-differentiate.

It has been reported in the literature incidentally PET detected ^18^F-FDG avid primary thyroid malignancies are generally a more aggressive variant of primary thyroid cancer which harbour a higher rate of unfavourable prognostic factors and are often less well differentiated[8]. In our case, the coexistent focus of metastatic colorectal adenocarcinoma within a primary poorly differentiated thyroid cancer somewhat complicates the issue, as both tumours are likely to be ^18^F-FDG avid.

To our knowledge there have been no cases of metastatic malignancy within a primary thyroid malignancy reported previously in the literature. As such, there is scant evidence in the literature regarding the most appropriate management strategy for such a patient. Conventional management of a primary thyroid malignancy usually involves total surgical thyroidectomy followed by radioiodine therapy to ablate the thyroid remnant, decrease risk of recurrence and enable adequate follow up using I-131 whole body scintigraphy and stimulated thyroglobulin levels [9-13].

In our case, prognosis is more likely to be dependent on the patient's metastatic rectal adenocarcinoma rather than the primary thyroid malignancy. Stage IV metastatic rectal adenocarcinoma portends a poor prognosis, with five year survival rates of between 4%–8%[14,15]. More specific to our case, in a review of 12 patients with metastatic rectal carcinoma to the thyroid between 1990 to 1993 by Fujita et al [3], in the one patient without metastases to any other organ but the thyroid, survival was just 4 years. Primary early stage non anaplastic thyroid carcinoma even if poorly differentiated has a better prognosis compared to metastatic rectal cancer. Poorly differentiated primary follicular thyroid carcinoma has been reported to have a 5 year survival rate of 63% in a recent published series of 40 patients[16].

As a result, the primary treatment focus in our patient was tailored towards treating metastatic rectal carcinoma and less so the primary thyroid malignancy. It is also of note, many poorly differentiated thyroid carcinomas are not particularly radioiodine avid and it is quite possible the impact of radioiodine therapy on reducing recurrence rates may be greatly diminished in this setting.

## Conclusion

Metastatic rectal carcinoma to the thyroid gland and in particular to a primary thyroid malignancy is uncommon and to our knowledge has not been reported. Prognosis is likely to be more dependent on underlying metastatic disease rather than the primary thyroid malignancy hence primary treatments should be tailored towards treating and controlling metastatic disease and less emphasis placed on the primary thyroid malignancy.

## Consent

Written informed consent was obtained from the patient for publication of this Case report and any accompanying images. A copy of the written consent is available for review by the Editor-in-Chief of this journal.

## Competing interests

The authors declare that they have no competing interests.

## Authors' contributions

MC conceived the idea, reported the PET scan, performed the literature search, provided the radiographic images and drafted the manuscript. MM administered chemotherapy, reviewed and revised manuscript, JS performed surgery, reviewed and revised manuscript, SS reported histopathology, provided histopathological images, reviewed and revised manuscript and DT treated patient, reviewed and revised manuscript. All authors have read and approved the final manuscript.
